# Natural History and Factors Influencing 90‐Day Clinical Outcomes and 1‐Year Recurrence of Thalamic Stroke

**DOI:** 10.1002/brb3.71755

**Published:** 2026-09-28

**Authors:** Huang Qiang, Cui Ying, Zhang Yongbo

**Affiliations:** ^1^ Department of Neurology, Beijing Friendship Hospital Capital Medical University Beijing China

**Keywords:** cerebral small vessel disease, clinical prognosis, recurrence, influencing factors, thalamic infarction

## Abstract

**Objective:**

To investigate the natural course of cerebral small vessel disease (CSVD) manifested as pure thalamic infarction (PTI) and identify predictors of 90‐day clinical prognosis and 1‐year recurrent major adverse cardiovascular/cerebrovascular events (MACE).

**Methods:**

This retrospective cohort study analyzed 251 consecutive thalamic infarction patients admitted between January 2016 and May 2024. Participants were classified into PTI (*n* = 187) or combined‐type thalamic infarction (CTI, *n* = 64). Primary endpoints included poor 90‐day clinical prognosis (modified Rankin Scale [mRS] ≥ 2) and 1‐year MACE (composite of stroke, acute myocardial infarction, and cardiovascular death). Intergroup comparisons utilized Pearson Chi‐square or Mann–Whitney U tests, with variables showing *p* ≤ 0.10 in univariate analysis entered into multivariate logistic regression models.

**Results:**

Compared with CTI, PTI patients demonstrated lower baseline National Institutes of Health Stroke Scale (NIHSS) scores (median 1 vs. 3, *p* < 0.001) and reduced prevalence of atrial fibrillation (2.7% vs. 12.5%), intracranial artery stenosis (ICAS; 48.1% vs. 76.6%), and early neurological deterioration (24.6% vs. 56.3%; all *p* < 0.05). The 90‐day poor prognosis rate was significantly lower in PTI (4.8% vs. 20.3%, *p* < 0.001), whereas 1‐year MACE rates did not differ between groups (12.3% vs. 15.6%, *p* = 0.497). Among PTI cases, territorial distributions comprised inferolateral (80.2%), tuberothalamic (13.4%), paramedian (including Percheron artery, 5.9%), and posterior choroidal arteries (0.5%). Tuberothalamic artery infarctions exhibited higher cognitive impairment rates (36.0%) than other subgroups. Multivariate analysis identified baseline NIHSS as an independent predictor of 90‐day poor prognosis (OR = 1.634, 95% CI 1.152–2.320, *p* = 0.006), while ICAS was associated with 1‐year MACE (OR = 3.908; 95% CI, 1.188–12.849; *p* = 0.025) in PTI patients.

**Conclusion:**

PTI presented favorable short‐term outcomes, consistent with initial stroke severity, yet carried a 12.3% annual MACE risk, particularly in patients with coexisting ICAS. These findings underscore the importance of addressing intracranial stenosis in secondary prevention strategies for CSVD.

## Abstract 2

Thalamic Infaction: PTI vs CTI Outcomes Retrospective cohort study 90‐day and 1‐year follow‐up PTI show better 90‐day prognosis but 1‐year MACE risk persist; 1‐year MACE is associated with MACE.

## 1 Background

The thalamus, a deep cerebral nucleus with multifaceted functional roles, receives its blood supply predominantly from perforating branches of the posterior cerebral artery. Despite the heterogeneous clinical manifestations of thalamic infarction, advances in neuroimaging modalities—particularly diffusion‐weighted imaging (DWI) and susceptibility‐weighted imaging (SWI)—have enhanced the timeliness and precision of diagnosing pure thalamic infarction (PTI; Bordes et al. 2020). This diagnostic reliability positions PTI as an ideal model for investigating cerebral small vessel disease (CSVD) or perforating artery disease (PAD). While prior studies have extensively characterized the vascular anatomy of thalamic territories and corresponding clinical phenotypes (Bordes et al. 2020; Chen et al. 2017; X. Wang et al. 2008), there remains a paucity of research comparing etiological patterns, clinical prognoses, and major adverse cardiovascular/cerebrovascular events (MACE) recurrence risks among PTI. To address this gap, this retrospective study analyzed 90‐day clinical outcomes and 1‐year follow‐up data from radiologically confirmed PTI cases (via DWI), aiming to inform personalized secondary prevention strategies for thalamic infarction patients.

## 2 Methods

### 2.1 Participants’ Eligibility and Inclusion

This retrospective cohort study consecutively enrolled thalamic infarction patients admitted to Beijing Friendship Hospital, Capital Medical University between January 2016 and May 2024. The stroke pathway was fully available for 7 days a week and 24 h, which was described in detail in our previous study (Huang et al. 2023). The inclusions and exclusions of candidate thalamic infarction patients were as follows. Inclusion Criteria: Age ≥ 18 years, regardless of sex; symptomatic acute thalamic infarction confirmed by DWI within 14 days of symptom onset; provision of written informed consent for stroke management. Exclusion criteria: non‐ischemic thalamic lesions (e.g., inflammatory, neoplastic, or cerebral venous thrombosis); chronic thalamic infarction showing isointense or hypointense signals on DWI; suspected thalamic infarction without DWI confirmation; loss to follow‐up or incomplete clinical documentation. Eligible patients received guideline‐directed revascularization therapies, including intravenous thrombolysis with alteplase (Actilyse, Boehringer Ingelheim) administered per 2014 Chinese Acute Ischemic Stroke Guidelines (Chinese Medical Association Neurological Credit Association, Department of Cerebrovascular Diseases in Chinese Medical Association Neurological Credit Committee 2015) and endovascular therapy with mechanical thrombectomy, stent retrieval, aspiration, or arterial thrombolysis, as per 2015 Chinese Neurovascular Intervention Guidelines (Chinese medical association neurological credit association, department of cerebrovascular diseases in Chinese medical association neurological credit Committee, department of neurovascular intervention collaborative group in Chinese medical association neurological credit Committee 2015). All patients were managed in dedicated stroke units with standard secondary prevention: including antiplatelet/anticoagulant agents, statins, and adjunctive therapies (*Ginkgo biloba* extract and butylphthalide injections) during hospitalization; vascular evaluation was using combined carotid ultrasound, transcranial Doppler (TCD), CT angiography (CTA), or MR angiography (MRA); and lesion of infarct was detected with MRI (with DWI and SWI). To ensure cohort homogeneity, inclusion required both clinical presentation (≤ 14 days from onset) and DWI‐confirmed acute thalamic infarction. And the accurate diagnosis of thalamic infarction comes from two independent individuals. One of them is the radiologist who provided the formal diagnostic report for thalamic infarction to patient. The author of this article, Huang Qiang, conducted a re‐examination of thalamic infarction images for all enrolled thalamic infarctions.

The study protocol (No. BFHHZS20240129) was approved by the Institutional Review Board of Beijing Friendship Hospital, Capital Medical University and was registered in the Medical Research Registration and Filing Information System (No. MR‐11‐24‐024437). In‐hospital management and data collection protocols followed methods detailed in our previous publication (Huang et al. 2023). The patient inclusion flowchart is presented in Supporting Information Figure S1.

### 2.2 Explanatory and Outcome Variables

Demographic data (including sex, age), baseline variables (severity of the stroke symptom measured as National Institutes of Health Stroke Scale [NIHSS]), medical history (hypertension, diabetes, dyslipidemia, coronary heart disease, atrial fibrillation, and prior history of transient ischemic attack [TIA]/stroke), current smoking (≥ 1 cigarette/day within 6 months), and heavy alcohol use (≥ 30 g/week within 6 months; Y. ‐L. Wang et al. 2010), as well as additional factors likely to be associated with clinical prognosis (such as territorial arteries of PTI; Bordes et al. 2020): tuberothalamic, paramedian (including Percheron artery, an abnormal variant of thalamic artery supply, with different clinical and radiological manifestations; Macedo et al. 2022), inferolateral, and posterior choroidal arteries; infarct characteristics: PTI versus combined type of thalamic infarction (CTI; X. Wang et al. 2008); PTI was confirmed by DWI with an ischemic region of thalamic nuclei at the basal ganglia level, which restricted to four groups of the thalamic blood supply territory (Bordes et al. 2020), while CTI was defined as not only thalamic infarction, but also acute cerebral infarction lesions in other brain regions confirmed by DWI (X. Wang et al. 2008). Concomitant arterial disease: cervical artery stenosis, intracranial arterial stenosis (ICAS), or peripheral artery stenosis (≥ 5 0% luminal narrowing); etiology of thalamic infarction: PAD versus non‐perforating mechanisms; treatment variables: with or without acute revascularization (intravenous thrombolysis or endovascular therapy); onset‐to‐door time (ODT), which was defined as time from symptom onset to initial hospital evaluation; post‐stroke sequelae including sensory deficits/thalamic pain (which was diagnosed via clinical history and medication records), cognitive impairment (measured with Montreal Cognitive Assessment, MoCA), psychiatric symptoms (measured with Hamilton Anxiety [HAMA] and depression [HAMD] Scales), and sleep disorders (measured with Pittsburgh Sleep Quality Index [PSQI] were assessed at the 90‐day follow‐up visit; Chen et al. 2017). Imaging markers: Infarct volume (mL), white matter hyperintensity severity, namely, mild (Fazekas score 0–2) versus moderate‐severe (Fazekas score 3–6) and cerebral microbleed (CMB) burden on SWI: Low (0–lesions) versus high (> 4 lesions; Chinese Medical Association Neurological Credit Association, Department of Cerebrovascular Diseases in Chinese Medical Association Neurological Credit Committee 2022); antithrombotic regimens: namely, monotherapy: aspirin, clopidogrel, cilostazol, ticagrelor, or anticoagulants (warfarin, rivaroxaban, edoxaban) and dual antiplatelet therapy (DAPT): aspirin + clopidogrel (or alternatives) for ≤ 21 days (Y. Wang et al. 2013), were collected in forms of case reports.

Outcome measures primary outcomes: Short‐term: Poor 90‐day clinical prognosis (defined as modified Rankin Scale [mRS] ≥ 2; mRS ranges from 0 [no symptoms] to 6 [death]). Long‐term: 1‐year recurrent MACE, including symptomatic myocardial infarction, stroke recurrence, and cardiovascular/cerebrovascular mortality. Secondary outcomes: Early neurological deterioration (increase of ≥ 1 point in NIHSS within 14 days, compared to baseline (National Institute of Neurological Disorders and Stroke rt‐PA Stroke Study Group 1995). Major hemorrhagic events (e.g., intracranial or gastrointestinal bleeding within 14 days. Intracranial bleeding was determined based on clinical symptoms plus follow up head imaging; while gastrointestinal bleeding was defined according to occult blood testing as well as related clinical manifestations, such as a spontaneous decrease in systolic blood pressure greater than 20 mmHg, an increase in heart rate greater than 20 beats per minute, a decrease in hemoglobin greater than 2 g/dL, or the need for treatment such as blood transfusion, intravenous vasoconstrictors, and/or invasive hemostatic surgery). Post‐thalamic infarction sequelae at 90 days: sensory deficits, thalamic pain, cognitive impairment, sleep disorders, and psychiatric symptoms.

### 2.3 Statistical Analysis

All analyses were performed using SPSS 22.0. Normally distributed continuous variables: Expressed as mean ± standard deviation (SD); compared via Student's *t*‐test. Non‐normally distributed variables: Reported as median (interquartile range, IQR); analyzed using Kruskal–Wallis test. Categorical variables: Presented as percentages (%); compared via Pearson χ^2^ test (or Fisher's exact test). Variables with *p* ≤ 0.10 in univariate analysis were included in logistic regression models. Results are reported as odds ratios (OR) with 95% confidence intervals (CI). Statistical significance was defined as two‐tailed *p* < 0.05.

## 3 Results

### 3.1 Baseline Characteristics of Enrolled Thalamic Infarction Patients

A total of 251 thalamic infarction patients were included, with 187 cases (74.5%) classified as PTI. As shown in Tables 1 and 2, compared to the combined‐type infarction (CTI) group, PTI patients exhibited significantly lower baseline NIHSS scores (median 1 vs. 3), reduced prevalence of atrial fibrillation (2.7% vs. 12.5%), peripheral artery disease (25.7% vs. 42.2%), cervical artery stenosis (34.2% vs. 54.7%), ICAS (48.1% vs. 76.6%), early neurological deterioration (24.6% vs. 56.3%), major hemorrhagic events (3.2% vs. 18.8%), and lower rates of post‐stroke sequelae including sleep disorders (13.4% vs. 25.0%), cognitive impairment (11.8% vs. 40.6%), psychiatric symptoms (3.2% vs. 17.2%), and 90‐day poor clinical prognosis (4.8% vs. 20.3%; all *p* < 0.05). No intergroup difference was observed in 1‐year MACE recurrence (12.3% vs. 15.6%, *p* = 0.497; Table 2). Among all patients, 100 (53.5%) received DAPT, aspirin + clopidogrel), and 18 (7.2%) experienced major bleeding events (11 intracranial, seven gastrointestinal; Table 1).

**TABLE 1:** Baseline characteristics of thalamic infarction cases included in this study.

	Total population (*n* = 251)	Pure thalamic infarction (*n* = 187)	Combined type of thalamic infarction (*n* = 64)	*p*
Age, years	63(56–70)	62(55–70)	65(57–72)	0.189
Female	75(29.9)	52(27.8)	23(35.9)	0.220
Medical history				
Hypertension	187(74.5)	136(72.7)	51(79.7)	0.270
Diabetes	124(49.4)	96(51.3)	28(43.8)	0.295
Dyslipidemia	172(68.5)	123(65.8)	49(76.6)	0.109
Coronary heart disease	42(16.7)	30(16.7)	12(18.8)	0.617
Atrial fibrillation	13(5.2)	5(2.7)	8 (12.5)	0.002
Peripheral artery stenosis	75(29.9)	48(25.7)	27(42.2)	0.013
Cervical artery stenosis	99(39.4)	64(34.2)	35(54.7)	0.004
Intracranial arterial stenosis	139(55.4)	90(48.1)	49(76.6)	<0.001
Prior stroke or TIA	61(24.3)	43(23.0)	18(28.1)	0.409
Current smoking	141(56.2)	108(57.8)	33(51.6)	0.389
Heavy alcohol use	115(24.2)	90 (48.1)	25(39.1)	0.209
NIHSS	2(1–3)	1(1–3)	3(2–4)	<0.001
Dual antiplatelet therapy	124(49.4)	100(53.5)	24(37.5)	0.027
Revascularization therapy	11(4.4)	6(3.2)	5(7.8)	0.120
Onset‐to‐door time, min	720(305–1440)	660(330–1440)	900(251–2880)	0.176
Imaging markers of cerebral small vessel disease				
Perforating artery disease	120(47.8)	115(61.5)	5(7.8)	<0.001
Volume of thalamic infarction, mL	0.309(0.130–0.570)	0.283(0.139–0.537)	0.403(0.104–0.866)	0.180
CMB count	0(0–2)	0(0–1)	0(0–3)	0.099
Low CMB burden	221(88.0)	165(88.2)	56(87.5)	0.876
Mild white matter hyperintensity	133(53.0)	108(57.8)	25(39.1)	0.010

*Note*: Unless otherwise stated, continuous and categorical data are presented as median (IQR) and percentage (%), respectively, with *p*‐values calculated using Mann–Whitney U and χ2 tests, respectively. TIA, transient ischemic attack.

Abbreviations: CMB, cerebral microbleed; MACE, major adverse cardiovascular/cerebrovascular events; mRS, modified Rankin scale; NIHSS, National Institutes of Health Stroke Scale.

**TABLE 2:** Clinical endpoints of thalamic infarction cases included in this study.

	Total population (*n* = 251)	Pure thalamic infarction (*n* = 187)	Combined type of thalamic infarction (*n* = 64)	*p*
Early neurological deterioration	82(32.7)	46(24.6)	36(56.3)	<0.001
Major hemorrhagic events	18(7.2)	6(3.2)	12(18.8)	<0.001
Sensory deficits sequelae	19(7.6)	15(8.0)	4(6.3)	0.644
Thalamic pain sequelae	73(29.1)	56(29.9)	17(26.6)	0.607
Sleep disorders sequelae	41(16.3)	25(13.4)	16(25.0)	0.030
Cognitive impairment sequelae	48(19.1)	22(11.8)	26(40.6)	<0.001
Psychiatric symptoms sequelae	17(6.8)	6(3.2)	11(17.2)	<0.001
Poor 90‐day clinical prognosis, mRS ≥ 2	22(8.8)	9(4.8)	13(20.3)	<0.001
1‐year recurrent MACE	33(13.1)	23(12.3)	10(15.6)	0.497

*Note*: Unless otherwise stated, continuous and categorical data are presented as median (IQR) and percentage (%), respectively, with *p*‐values calculated using Mann–Whitney U and χ2 tests, respectively.

Abbreviations: CMB, cerebral microbleed; MACE, major adverse cardiovascular/cerebrovascular events; mRS, modified Rankin scale; NIHSS, National Institutes of Health Stroke Scale; TIA, transient ischemic attack.

### 3.2 Territorial Distribution and Outcomes in PTI Patients

Analysis of 187 PTI cases revealed the following territorial artery involvement (Table 3): inferolateral thalamic artery (80.2%, 150/187), tuberothalamic artery (13.4%, 25/187), paramedian thalamic artery (including Percheron artery, 5.9%, 11/187), and posterior choroidal artery (0.5%, 1/187). Notably, tuberothalamic artery infarctions demonstrated a higher incidence of cognitive impairment sequelae (*p* < 0.001), compared to other subgroups, while other outcomes showed no significant differences (*p* > 0.05; Table 3). The PTI cohort had a median age of 62 (55–70) years, 27.8% female, baseline NIHSS of 1 (1–3), 90‐day poor prognosis rate of 4.8% (9/187), and 1‐year MACE recurrence rate of 12.3% (23/187; Table 4).

**TABLE 3:** Territorial distribution and outcomes in pure thalamic infarction patients.

	Tuberothalamic artery(*n* = 25)	Paramedian thalamic artery(*n* = 11)	Inferolateral thalamic artery(*n* = 150)	Posterior choroidal artery(*n* = 1)	*p*
Early neurological deterioration	7(28.0%)	5(45.5%)	34(22.7%)	0(0)	0.334
Major hemorrhagic events	1(4.0%)	1(9.1%)	4(2.7%)	0(0)	0.239
Sensory deficits sequelae	1(4.0%)	1(9.1%)	13(8.7%)	0(0)	0.772
Thalamic pain sequelae	6(24.0%)	4(36.4%)	46(30.7%)	0(0)	0.782
Sleep disorders sequelae	5(20.0%)	2(18.2%)	18(12.0%)	0(0)	0.516
Cognitive impairment sequelae	9(36.0%)	3(27.3%)	10(6.7%)	0(0)	<0.001
Psychiatric symptoms sequelae	0(0)	1(9.1%)	5(3.3%)	0(0)	0.469
Poor 90‐day clinical prognosis, mRs > 1	1(4.0%)	2(18.2%)	6(4.0%)	0(0)	0.177
1‐year recurrent MACE	4(16.0%)	3(27.3%)	16(10.7%)	0(0)	0.271

*Note*: Unless otherwise specified, categorical data are expressed as a percentage (%). When the theoretical frequency of any cell is less than 1, Fisher's exact test (rather than chi square test) is used to calculate the *p*‐value.

Abbreviations: MACE, major adverse cardiovascular/cerebrovascular events; mRS, modified Rankin scale.

**TABLE 4:** Baseline characteristics of PTI cases included in this study.

	Total PTI population(*n* = 187)	90‐day clinical prognosis Good (*n* = 178) Poor (*n* = 9)*p*			1‐year recurrent MACE No (*n* = 164) Yes (*n* = 23)*p*		
Age, years	62(55–70)	63(55–70)	59(57–69)	0.067	62(55–70)	61(56–71)	0.962
Female	52(27.8)	49(27.5)	3(33.3)	0.709	45(27.4)	7(30.4)	0.764
Medical history							
Hypertension	136(72.7)	128(71.9)	8(88.9)	0.225	116(70.7)	20(87.0)	0.102
Diabetes	96(51.3)	89(50.0)	7(77.8)	0.104	80(48.8)	16(69.6)	0.062
Dyslipidemia	123(65.8)	115(64.6)	8(88.9)	0.102	102(62.2)	21(91.3)	0.006
Coronary heart disease	30(16.0)	27(15.2)	3(33.3)	0.189	25(15.2)	5(21.7)	0.427
Atrial fibrillation	5(2.7)	5(2.8)	0(0)	0.479	5(3.0)	0(0)	0.249
Peripheral artery stenosis	48(25.7)	43(24.2)	5(55.6)	0.051	38(23.2)	10(43.5)	0.037
Cervical artery stenosis	64(34.2)	60(33.7)	4(44.4)	0.515	53(32.3)	11(47.8)	0.142
Intracranial arterial stenosis	90(48.1)	84(47.2)	6(66.7)	0.251	73(44.5)	17(73.9)	0.008
Prior stroke or TIA	43(23.0)	40(22.5)	3(33.3)	0.469	33(20.1)	10(43.5)	0.013
Current smoking	108(57.8)	103(57.9)	5(55.6)	0.891	92(56.1)	16(69.6)	0.221
Heavy alcohol use	90(48.1)	86 (48.3)	4(44.4)	0.820	79 (48.2)	11(47.8)	0.975
NIHSS	1(1–3)	1(1–3)	7(2–10)	<0.001	1(1–3)	2(1–3)	0.030
Dual antiplatelet therapy	100(53.5)	99(55.6)	1(11.1)	0.006	92(56.1)	8(34.8)	0.055
Revascularization therapy	6(3.2)	5(2.8)	1(11.1)	0.270	5(3.0)	1(4.3)	0.741
**Imaging markers of cerebral small vessel disease**							
Perforating artery disease	115(61.5)	110(61.8)	5(55.6)	0.709	103(62.8)	12(52.2)	0.326
Volume of thalamic infarction, mL	0.283(0.139–0.537)	0.278(0.139–0.531)	0.450(0.06–0.936)	0.667	0.266(0.133–0.534)	0.417(0.144–0.559)	0.304
CMB count	0(0–1)	0(0–2)	0(0–0)	0.396	0(0–1)	0(0–3)	0.216
Low CMB burden	165(88.2)	156(87.6)	9(100.0)	0.128	145(88.4)	20(87.0)	0.839
Mild white matter hyperintensity	108(57.8)	104(58.4)	4(44.4)	0.411	99(60.4)	9(39.1)	0.054
**Clinical endpoints**							
Early neurological deterioration	46(24.6)	40(22.5)	6(66.7)	0.006	38(23.2)	8(34.8)	0.226
Major hemorrhagic events	6(3.2)	5(2.8)	1(11.1)	0.270	5(3.0)	1(4.3)	0.751
Sensory deficits sequelae	15(8.0)	15(8.4)	0(0)	0.214	14(8.5)	1(4.3)	0.456
Thalamic pain sequelae	56(29.9)	54(30.3)	2(22.2)	0.594	49(29.9)	7(30.4)	0.956
Sleep disorders sequelae	25(13.4)	20(11.2)	5(55.6)	0.002	19(11.6)	6(26.1)	0.056
Cognitive impairment sequelae	22(11.8)	19(10.7)	3(33.3)	0.079	18(11.0)	4(17.4)	0.371
Psychiatric symptoms sequelae	6(3.2)	4(2.2)	2(22.2)	0.022	5(3.0)	1(4.3)	0.751

*Note*: Unless otherwise stated, continuous and categorical data are presented as median (IQR) and percentage (%), respectively, with *p*‐values calculated using Mann–Whitney U and χ2 tests, respectively.

Abbreviations: CMB, cerebral microbleed; MACE, major adverse cardiovascular/cerebrovascular events; mRS, modified Rankin scale; NIHSS, National Institutes of Health Stroke Scale; PTI, pure thalamic infarction; TIA, transient ischemic attack.

### 3.3 Multivariate Analysis of Prognostic Factors

Baseline characteristics of all thalamic infarction (*n* = 251) and PTI patients (*n* = 187) were shown in Tables 1 and 4, respectively. Variables with *p* ≤ 0.10 in univariate analysis for 90‐day poor prognosis and 1‐year MACE recurrence were included in logistic regression models to detect prognostic factors, respectively. The detailed result of the logistic regression models are shown in Tables 5 and 6.

**TABLE 5:** Risk factors of primary outcomes in all eligible patients.

	Odds ratio	95% confidence interval	*p*
**Risk factors of poor 90‐day clinical prognosis in all eligible patients**			
Dyslipidemia	2.638	0.271–25.720	0.404
Atrial fibrillation	1.655	0.038–72.254	0.794
Peripheral artery stenosis	15.961	1.018–250.145	0.049
Cervical artery stenosis	0.059	0.003–1.314	0.074
Intracranial arterial stenosis	0.451	0.026–7.873	0.585
NIHSS	2.141	1.473–3.106	<0.001
Dual antiplatelet therapy	0.076	0.007–0.810	0.033
Perforating artery disease	0.694	0.057–8.510	0.775
CMB count	1.324	1.035–1.693	0.025
Mild White matter hyperintensity	4.938	0.647–37.688	0.124
Early neurological deterioration	2.298	0.308–17.145	0.417
Major hemorrhagic events	22.319	1.418–351.388	0.027
**Risk factors of 1‐year recurrent MACE in all eligible patients**			
Dyslipidemia	3.190	0.733–13.893	0.122
Peripheral artery stenosis	0.885	0.278–2.824	0.837
Cervical artery stenosis	0.840	0.261–2.704	0.771
Intracranial arterial stenosis	2.829	0.771–10.383	0.117
Prior stroke or TIA	2.803	1.022–7.692	0.045
NIHSS	1.027	0.906–1.164	0.677
Dual antiplatelet therapy	0.758	0.267–2.147	0.601
Volume of thalamic infarction, mL	0.907	0.419–1.962	0.804
CMB count	1.053	1.002–1.106	0.041
Mild White matter hyperintensity	1.317	0.485‐3.573	0.589
Early neurological deterioration	0.546	0.163–1.831	0.327
Major hemorrhagic events	2.503	0.447–14.015	0.297
Sleep disorders sequelae	3.518	0.934–13.250	0.063
Cognitive impairment sequelae	0.361	0.080–1.632	0.186
Psychiatric symptoms sequelae	0.430	0.047–3.945	0.455
Poor 90‐day clinical prognosis	23.769	4.448–127.008	<0.001

Abbreviations: CMB, cerebral microbleed; MACE, major adverse cardiovascular/cerebrovascular events; NIHSS, National Institutes of Health Stroke Scale; TIA, transient ischemic attack.

**TABLE 6:** Risk factors of primary outcomes in pure thalamic infarction patients.

	Odds ratio	95% confidence interval	*p*
**Risk factors of poor 90‐day clinical prognosis in PTI**			
Age, years	1.054	0.956–1.163	0.289
NIHSS	1.634	1.152–2.320	0.006
Early neurological deterioration	1.528	0.198–11.768	0.684
Peripheral artery stenosis	5.350	0.450–63.574	0.184
Dual antiplatelet therapy	0.208	0.020–2.124	0.185
Sleep disorders sequelae	7.257	0.621–84.783	0.114
Cognitive impairment sequelae	0.094	0.001–10.104	0.322
Psychiatric symptoms sequelae	2.314	0.038–139.842	0.688
**Risk factors of 1‐year recurrent MACE in PTI**			
Diabetes	2.333	0.766–7.105	0.136
Dyslipidemia	4.829	0.990–23.554	0.051
NIHSS	1.171	0.955–1.437	0.129
Peripheral artery stenosis	0.610	0.191–1.953	0.405
Intracranial arterial stenosis	3.908	1.188–12.849	0.025
Prior stroke or TIA	1.719	0.616–4.795	0.300
Dual antiplatelet therapy	0.439	0.153–1.255	0.125
Mild White matter hyperintensity	1.788	0.658–4.860	0.255
Sleep disorders sequelae	1.456	0.359–5.907	0.599

Abbreviations: MACE, major adverse cardiovascular/cerebrovascular events; NIHSS, National Institutes of Health Stroke Scale; PTI, pure thalamic infarction; TIA, transient ischemic attack.

90‐day poor prognosis: Logistic regression incorporating variables with *p* ≤ 0.10 identified four independent risk factors: Peripheral artery disease (OR = 15.961; 95% CI 1.018–250.145; *p* = 0.049); CMB burden (OR = 1.324; 95% CI 1.035–1.693; *p* = 0.025); baseline NIHSS (OR = 2.141; 95% CI 1.473–3.106; *p* < 0.001); major hemorrhage (OR = 22.319; 95% CI 1.418–351.388; *p* = 0.027); DAPT was protective (OR = 0.076; 95% CI 0.007–0.810; *p* = 0.033).

1‐year mace recurrence: Significant predictors included: CMB burden (OR = 1.053; 95% CI 1.002–1.106; *p* = 0.041); prior stroke/TIA history (OR = 2.803; 95% CI 1.022–7.692; *p* = 0.045); and 90‐day poor prognosis (OR = 23.769; 95% CI 4.448–127.008; *p* < 0.001).

PTI‐specific analysis: In the PTI subgroup (*n* = 187): Baseline NIHSS independently predicted 90‐day poor prognosis (OR = 1.634; 95% CI 1.152–2.320; *p* = 0.006). ICAS was associated with 1‐year MACE recurrence (OR = 3.908; 95% CI 1.188–12.849; *p* = 0.025).

## 4 Discussion

Our study demonstrates that PTI generally exhibits favorable short‐term outcomes, with a low incidence of poor 90‐day clinical prognosis (4.8%). Baseline NIHSS independently predicts adverse clinical outcomes. Only 11 patients (4.4%) included in this study received revascularization therapy (mostly intravenous thrombolysis) due to the long ODT. The mean ODT of the total population was 720 (305–1440) min (Table 1), and the ODT of the vast majority of thalamic stroke patients exceeded the conventional time window of intravenous thrombolysis by 4.5 h. The 1‐year recurrence rate of MACE in PTI patients was 12.3%, and PTI with ICAS could significantly increase this risk. Among all 251 enrolled thalamic infarction patients, DAPT reduced the 90‐day poor prognosis risk, while high CMB burden, elevated baseline NIHSS, and major hemorrhagic events were independent risk factors. High CMB burden and poor 90‐day clinical prognosis predicted 1‐year MACE recurrence.

Antiplatelet therapy remains the cornerstone of secondary prevention for symptomatic CSVD, including PTI. Our findings suggest that a 21‐day DAPT regimen reduces 90‐day poor prognosis risk in PTI patients. This result aligns with the POINT trial, where DAPT for 90 days significantly benefited patients with radiologically confirmed infarcts (OR = 0.64, 95% CI 0.45–0.91; Johnston et al. 2018). Current guidelines, informed by the CHANCE (Y. Wang et al. 2013) and POINT (Johnston et al. 2018) trials, recommend short‐term DAPT (21–30 days) followed by monotherapy (e.g., aspirin, clopidogrel, or cilostazol). Recent evidence from the INSPIRES trial (*n* = 5126) further supports DAPT's superiority over monotherapy in reducing 90‐day stroke recurrence across all CSVD burden subgroups (Li et al. 2025). A 2024 study highlighted that ischemic event risks outweigh bleeding risks in CSVD patients, suggesting net clinical benefits from appropriate antithrombotic strategies (Tanaka et al. 2024). A network meta‐analysis of 24 RCTs (*n* = 47,507) identified cilostazol monotherapy as optimal for preventing MACE (SUCRA 90.0%, OR = 0.66 vs. aspirin, 95% CI 0.49–0.89; Feng et al. 2025). The PACIFIC‐STROKE trial reported the safety and potential efficacy of the factor XIa inhibitor asundexian in high‐bleeding‐risk CSVD patients with high CMB burden (Balali et al. 2024). Emerging agents like cilostazol and asundexian show promise in balancing efficacy and bleeding risks, though their long‐term safety in CSVD subtypes (e.g., ICAS‐comorbid) requires further validation (Feng et al. 2025; Sharma et al. 2026).

Bleeding complications remain a critical limitation of antithrombotic therapy in CSVD. Current risk stratification integrates imaging biomarkers (e.g., CMB burden, Best et al. 2021; white matter hyperintensity severity, Best et al. 2020) with some clinical variables. The BAT2 study (*n* = 5250) demonstrated that total SVD score (0–4) independently predicts bleeding: a score of 4 increased major bleeding risk by 5.47‐fold and intracranial hemorrhage risk by 9.29‐fold (Tanaka et al. 2024). The MICON consortium model (*n* = 15,766) achieved a C‐index of 0.73 for 5‐year intracranial hemorrhage prediction (Best et al. 2021). The 2025 BAT2 score, incorporating age, renal dysfunction, hypertension, CMB, lacunes, and antithrombotic agents, showed a C‐index of 0.75 (Miwa et al. 2025). Additionally, high basal ganglia perivascular spaces (≥ 11) were linked to elevated bleeding risk (Iwamoto et al. 2026). These tools enable data‐driven decision‐making for personalized antithrombotic strategies.

ICAS significantly increased MACE recurrence in PTI patients. This finding could reflect a greater overall cerebrovascular disease burden and more severe chronic hypoperfusion leading to higher recurrent ischemic risk associated with ICAS. On the one hand, it may mean that extended DAPT may benefit high ischemic stroke burden patients with ICAS. However, the SAMMPRIS trial previously demonstrated DAPT's non‐inferiority to stenting in ICAS‐related stroke prevention (Derdeyn et al. 2014). While a 2025 MRI/MRA study (*n* = 1206) found ICAS independently associated with lacunes (OR = 2.95, 95% CI 1.97–4.43, *p* < 0.001), white matter hyperintensities (β = 0.333, *p* < 0.001), and cortical atrophy (β = −0.011, *p* < 0.001) but not with CMBs (Pan et al. 2025). ICAS‐related lacunes predominantly involved the basal ganglia, while non‐ICAS lacunes localized to the centrum semiovale; and ICAS also correlated with bilateral pyramidal tract/thalamic radiation white matter lesions and severe cortical atrophy (Pan et al. 2025). A recent Japanese study further linked blood pressure metrics to both lacunar infarcts and ICAS (Salman et al. 2022), underscoring ICAS's role in CSVD pathogenesis. The above research findings suggest that the role of ICAS should not be overlooked when assessing the overall disease burden of CSVD. On the other hand, in terms of neuronal protection in symptomatic ICAS, especially in temporary cerebral ischemia and reperfusion models, the efficacy of novel neuroprotective agents such as nelonemdaz without serious side effects on ICAS deserves further exploration (Hong et al. 2022).

This single‐center cohort study from Beijing has inherent limitations: (1) Moderate sample size may reduce statistical power and introduce selection bias, though our PTI cohort (*n* = 187) represents one of the largest reported; (2) regional homogeneity (Han Chinese population) limits generalizability; (3) retrospective design precludes causal inference. And we also acknowledge that some clinical endpoints (such as PTI, CTI, and major hemorrhagic events) and post‐thalamic infarction sequelae (such as sensory deficits, and thalamic pain) at 90 days were captured only via self‐report, which may introduce common method variance. However, on the one hand, cerebral infarction in the thalamus with fixed location and blood supply has a very high recognition rate in DWI imaging; on the other hand, in order to ensure objectivity and accuracy to the greatest extent possible under existing data conditions, we have implemented a relatively strict definition of practical operations. The accurate diagnosis of thalamic infarction comes from two independent individuals. One of them is the radiologist who provided the formal diagnostic report for patients. The author of this article, Huang Qiang, conducted a re‐examination of thalamic infarction images for all enrolled thalamic infarctions. Nevertheless, the methodology remains robust, offering valuable insights for CSVD (including PTI) management.

## 5 Conclusion

PTI, a CSVD hallmark, demonstrates low short‐term morbidity, but significant long‐term MACE risks, particularly for PTI patients with ICAS.

## Author Contributions

**Huang Qiang**: methodology, data curation, formal analysis, Writing – original draft, validation. **Zhang Yongbo**: methodology, investigation, formal analysis, writing – review and editing, resources. **Cui Ying**: data curation, investigation, formal analysis.

## Funding

The authors have nothing to report.

## Informed Consent

Informed consent was obtained from every participant.

## Conflicts of Interest

The authors declare no conflicts of interest.

## Supporting information




**Supporting Information Figure S1**: brb371755‐sup‐0001‐FigureS1.docx

## Data Availability

All the datasets used during the current study would be available from the corresponding author upon reasonable request.
