# Supplementary material for: Natural History and Factors Influencing 90‐Day Clinical Outcomes and 1‐Year Recurrence of Thalamic Stroke
Source: Brain Behav. 2026 Sep 28;16(10):e71755. doi: 10.1002/brb3.71755 (PMC13620292; doi:10.1002/brb3.71755)
Supplement: Supplementary file 1 [file BRB3-16-e71755-s001.docx]

Research flow chart for this study

(1) 12 cases without the consent of patients or their families;

(2) 29 cases with chronic thalamic infarction;

(3) 9 cases had incomplete data.

A total of 251 cases with the diagnosis of pure or combined “thalamic infarction” confirmed by DWI were detected.

A total of 301 cases with the diagnosis of “thalamic infarction” between January 2016 and May 2024 were detected.

64 combined thalamic infarction cases were included.

187 pure thalamic infarction cases were included.
